# Retraction: Global incidence and mortality of childhood leukemia and its relationship with the Human Development Index

**DOI:** 10.1371/journal.pone.0339086

**Published:** 2025-12-17

**Authors:** The PLOS One Editors

The *PLOS One* Editors retract this article [1] due to concerns about potential manipulation of the publication process. These concerns call into question the validity and provenance of the reported results and compliance with PLOS policies. We regret that the issues were not identified prior to the article’s publication.

All authors did not agree with the retraction.
